# Comparison and development of a metagenomic next-generation sequencing protocol for combined detection of DNA and RNA pathogens in cerebrospinal fluid

**DOI:** 10.1186/s12879-022-07272-y

**Published:** 2022-04-01

**Authors:** Hanfang Jiang, Zhihao Xing, Xiaorong Liu, Qiang Chai, Zefeng Xin, Chunqing Zhu, Ruihong Lin, Xuwen Deng, Dong Cui, HongDan Gao, Dongli Ma

**Affiliations:** 1 Institute of Pediatrics, Clinical Laboratory, Shenzhen Children’s Hospital, Shenzhen, Guangdong People’s Republic of China; 2 Institute of Pediatrics, Shenzhen Children’s Hospital, Shenzhen, Guangdong People’s Republic of China; 3 Medical Technology, Bengbu Medical College, Bengbu, Anhui People’s Republic of China

**Keywords:** Encephalitis, Next-generation sequencing, Metagenomic diagnosis

## Abstract

**Background:**

The purpose of this study was to evaluate different pretreatment, extraction, amplification, and library generation methods for metagenomic next-generation sequencing (mNGS) of cerebrospinal fluid (CSF) and to develop an efficient procedure for the simultaneous detection of DNA and RNA pathogens.

**Methods:**

We generated thirteen mock CSF samples with four representative pathogens of encephalitis. Each sample was subjected to ten different methods by varying sample pretreatment/nucleic acid extraction (microbial DNA, total DNA, total NA, total RNA, Whole Transcriptome Amplification (WTA)) and library generation (Illumina or NEB). Negative extraction controls (NECs) were used for each method variation.

**Results:**

We found that the quality of mNGS sequencing reads was higher from the NEB kit for library generation. Microbial DNA and total RNA increased microbial deposition by depleting the host DNA. Methods total NA and total RNA can detect gram-positive, gram-negative, RNA and DNA pathogens. We applied mNGS, including total NA and NEB library generation, to CSF samples from five patients diagnosed with infectious encephalitis and correctly determined all pathogens identified in clinical etiological tests.

**Conclusions:**

Our findings suggested that total nucleic acid extraction combined with NEB library generation is the most effective mNGS procedure in CSF pathogen detection. The optimization of positive criteria and databases can improve the specificity and sensitivity of mNGS diagnosis.

*Trial registration:* Chinese Clinical Trial Registry, ChiCTR1800015425 (29/03/2018), https://www.chictr.org.cn/edit.aspx?pid=26292&htm=4.

**Supplementary Information:**

The online version contains supplementary material available at 10.1186/s12879-022-07272-y.

## Background

Infectious encephalitis is a serious infectious disease of the central nervous system caused by viruses, bacteria, fungi or parasites. The high incidence rate and high mortality rate of encephalitis have made it a global public health concern. The prognosis of children with infectious encephalitis is poor; most of them cannot be cured after discharge and have persistent long-term neurological sequelae or even epilepsy [1]. Infectious encephalitis is the main cause of death in children under 5 years old [2].

The known pathogens of infectious encephalitis in children are mainly viruses and bacteria. Viruses include enterovirus, herpes simplex virus, human paraviruses and arboviruses. Bacteria include *Streptococcus pneumoniae, Escherichia coli, Streptococcus agalactiae, Haemophilus influenzae*, and *Neisseria meningitides *[3, 4]. The spectrum of infectious encephalitis in children covers a wide range, and there are rare or unknown pathogens causing disease. However, due to the limitations of microbial cultivation or PCR of cerebrospinal fluid, 60% of children with encephalitis cannot be diagnosed [5]. How to determine the pathogen causing infectious encephalitis has become an urgent problem. In 2014, the Chiu group applied metagenomics to diagnose a case of leptospiral encephalitis, which was the first case in the world that was successfully diagnosed by metagenomics [6]. In 2020, Chinese scientists established metagenomics technology to diagnose tuberculous encephalitis in an HIV-negative population [7]. In recent years, successful cases [8–13] or clinical trials [14–21] of metagenomics in the diagnosis of infectious encephalitis have gathered increasing interest in scientific research and transformation.

Metagenomics targets the mixed genome sequences or 16S rRNA sequences of all microorganisms in the environment [22]. Metagenomic analysis can obtain all the microbial genome sequence information in the samples, which is helpful to detect pathogens without bias. The process of detecting encephalitis pathogens by metagenomic sequencing includes sample pretreatment, nucleic acid extraction, library generation, sequencing, and bioinformatic analysis. The methods of sample pretreatment and nucleic acid extraction determine the sensitivity of pathogen detection [15]. In addition, sample pretreatment, nucleic acid extraction and library generation are labor intensive, which are the speed-limiting steps of the process [23].

At present, there are two problems in metagenomic diagnosis for infectious encephalitis. First, due to the low content of total nucleic acids in cerebrospinal fluid and contamination of the host background, RNA virus detection is difficult. Second, the separate operations to detect DNA microorganisms and RNA microorganisms are time-consuming and costly [17, 20, 24]. Our work aims to develop a single nucleic acid extraction step and a single library generation for the detection of DNA and RNA pathogens in cerebrospinal fluid. The modified process combined with downstream bioinformatic analysis and positive result interpretation will comprehensively detect DNA and RNA pathogens and reveal the etiology of infectious encephalitis in pediatric patients.

## Methods

### Construction of pathogen-positive cerebrospinal fluid and negative control

Four pathogenic microorganisms, *Streptococcus pneumoniae* (SP), *Escherichia coli* (E. coli), Epstein Barr virus (EBV), and enterovirus 71 (EV71), were obtained from an in vitro culture. The colony forming units (CFU/ml) of *Streptococcus pneumoniae* and *Escherichia coli* were identified by plate colony-counting methods. The copy number (copy/ml) of Epstein Barr virus and EV71 was identified by digital PCR. The residual CSF after clinical examination of pediatric patients without infections was collected. The pathogens were added to “normal” CSF to make of infectious encephalitis CSF samples 1 to 13. The negative control (NEC) was ultrapure water. The sample information is shown in Additional file 1: Table S1.

### Sample pretreatment/nucleic acid extraction

**Fig. 1:**
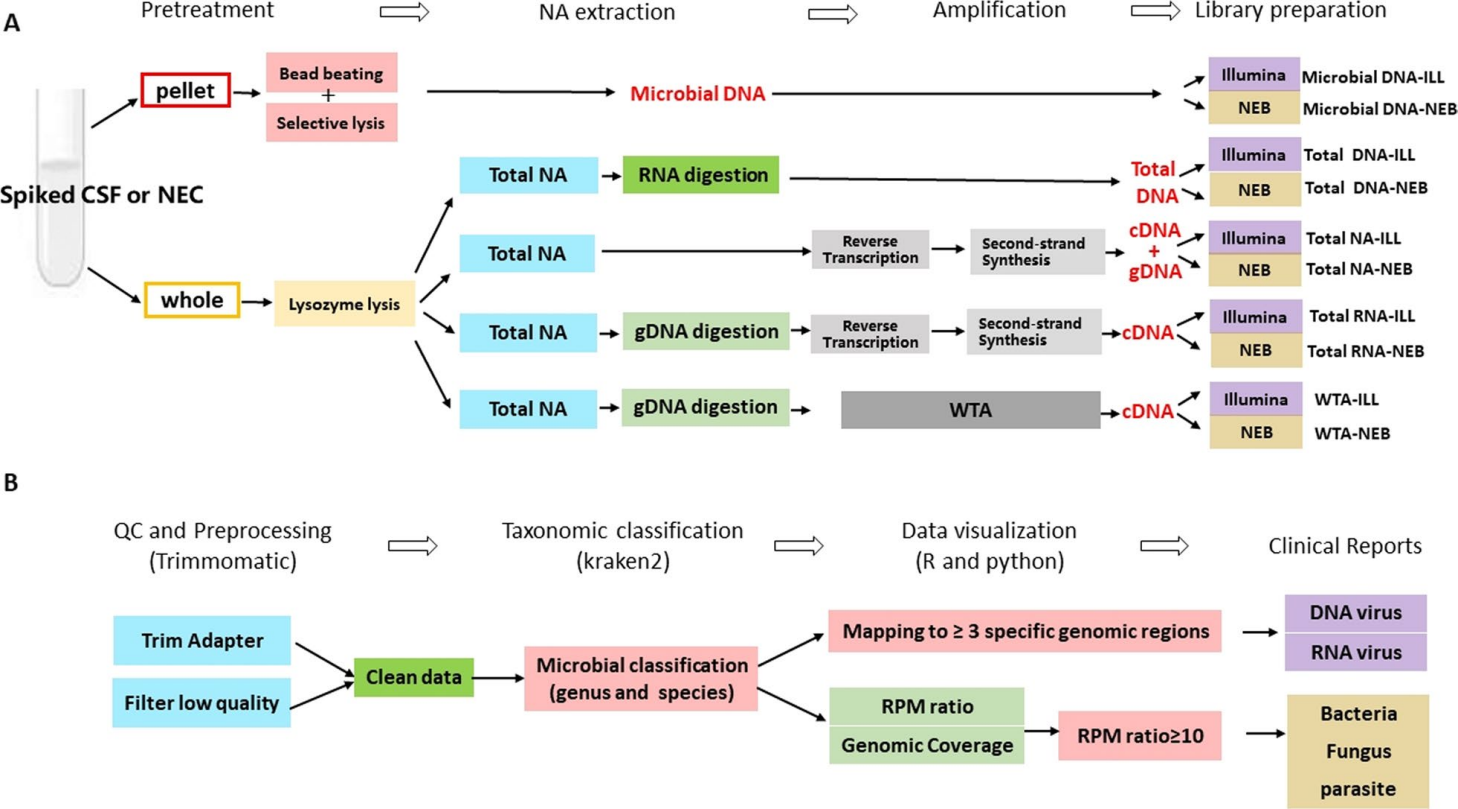
Development of the metagenomic next-generation sequencing protocol. **A** Overview of the method comparison study. There are ten methods named Microbial DNA-ILL, Microbial DNA-NEB, Total DNA-ILL, Total DNA-NEB, Total NA-ILL, Total NA-NEB, Total RNA-ILL, Total RNA-NEB, WTA-ILL and WTA-NEB. Microbial DNA-ILL: firstly the supernatant of CSF sample was removed, the microbial DNA was extracted after selective lysis then library was constructed with Illumina kit. Microbial DNA-NEB: firstly the supernatant of CSF sample was removed, the microbial DNA was extracted after selective lysis then the library was constructed with NEB kit. Total DNA-ILL: After the whole CSF sample was treated by lysozyme, the total DNA was extracted and retained then the library was constructed with Illumina kit. Total DNA-NEB: After the whole CSF sample was treated by lysozyme, the total DNA was extracted and retained then the library was constructed with NEB kit. Total NA-ILL: After the whole CSF sample was treated by lysozyme, the total nucleic acid was extracted then the library was constructed with Illumina kit. Total NA-NEB: After the whole CSF sample was treated by lysozyme, the total nucleic acid was extracted then the library was constructed with NEB kit. Total RNA-ILL: After the whole CSF sample was treated by lysozyme, the total RNA was extracted and retained by digesting genomic DNA then the library was constructed with Illumina kit. Total RNA-NEB**:** After the whole CSF sample was treated by lysozyme, the total RNA was extracted and retained by digesting genomic DNA then the library was constructed with NEB kit. WTA-ILL: After the whole CSF sample was treated by lysozyme, the total RNA was obtained and amplified by WTA kit then the library was constructed with Illumina kit. WTA-NEB: After the whole CSF sample was treated by lysozyme, the total RNA was obtained and amplified by WTA kit then the library was constructed with NEB kit. Each sample (sample1-14) had ten subsamples according to the ten methods. NA, nucleid acid; gDNA, genomic DNA; WTA, whole-transcriptome amplification using REPLI-g WTA Single Cell Kit; RT-PCR, reverse transcription PCR.B. Overview of Bioinformatic analysis. The raw sequencing data was preprocessed to gain clean data. Kraken2 was used to perform microbial classification. The positive cutoff of virus and nonviral pathogens were described in “Analysis of Results” in “Method”

Five types of sample pretreatment/nucleic acid extraction were named “Microbial DNA”, “Total DNA”, “Total NA”, “Total RNA” and “WTA”. The steps of each process are shown in Fig. 1A. The details are described as follows:

#### Microbial DNA

The host cells in the samples were gently destroyed by 0.033% saponin to release host DNA [25], which was digested by subsequent DNaseI and removed. Then, the QIAamp DNA Microbiome Kit (QIAamp, Germany) was used to extract microbial DNA.

#### Total DNA

The samples were treated with 60 mg/ml lysozyme and 1% Triton X-100 at 37 °C for 30 min. Then, the QIAamp DNA Mini Kit was used to extract total DNA.

#### Total NA

The samples were treated with 60 mg/ml lysozyme and 1% Triton X-100 at 37 °C for 30 min. Then, the EasyPure® RNA Kit (Transgene, Beijing, China) was used to extract total nucleic acids, including RNA and DNA. Total NA was reverse transcribed by a "Transcriptor first strand cDNA synthesis kit" (Roche, Switzerland). The mRNA second strand was synthesized by "mRNA Second Strand Synthesis Module" (NEB, USA). Finally, cDNA and gDNA (genomic DNA) was used as the template for library generation.

#### Total RNA

The samples were treated with 60 mg/ml lysozyme and 1% Triton X-100 at 37 °C for 30 min. Then, the EasyPure® RNA Kit was used to extract total nucleic acids, including RNA and DNA. Then, DNaseI was added to remove DNA. The remaining RNA was reverse transcribed by a "Transcriptor first strand cDNA synthesis kit". The mRNA second strand was synthesized by "mRNA Second Strand Synthesis Module". Finally, cDNA was used as the template for library generation.

#### WTA

The samples were treated with 60 mg/ml lysozyme and 1% Triton X-100 at 37 °C for 30 min. Then, the EasyPure® RNA Kit was used to extract total nucleic acids, including RNA and DNA. Then, DNaseI was added to remove DNA. cDNA was obtained by a REPLI-g WTA Single Cell Kit (QIAamp, Germany).

### Library generation

There were two library generation kits used: Illumina and NEB.

#### Illumina

According to the Illumina Nextera® XT Library Prep kit (Illumina, USA), the nucleic acids were quantified by Qubit™ 3.0 Fluorometer and diluted to 0.2 ng/µL. Five microliters of DNA was added to 10 μL of tagged DNA buffer and 5 μL of amplicon tag mix in a 96-well PCR plate, gently mixed and placed on a thermal circulator (TC) at 55 °C for 5 min followed by 10 °C for a short time. Five microliters of neutralization buffer were added to each sample well; the plate was centrifuged for 1 min and incubated at room temperature for 5 min. The samples were indexed with 5 μl of i5 and i7 index adapters, and 15 μl of Nextera PCR main mixture was added to each sample well and mixed gently. Then, the plate was put into a TC: 72 °C for 3 min; 95 °C for 30 s; followed by 12 cycles at 95 °C for 10 s, 55 °C for 30 s, and 72 °C for 30 s; 72 °C for 5 min and maintained at 10 °C. The library was purified with Agencourt AMPure XP beads and then washed twice with 80% ethanol and Illumina resuspension buffer. Qubit, agarose gel electrophoresis and qPCR were used for quality control.

#### NEB

According to the NEBNext® Ultra TM II DNA Library Prep Kit (NEB, USA), the extracted nucleic acids were quantified by Qubit™ 3.0 Fluorometer and then sheared to 300 bp fragments by covariate M220. Fifty microliters of fragmented nucleic acid was added to 3 µl of End Prep Enzyme Mix and 7 µl of End Prep Reaction Buffer. The mixture was mixed well. PCR was performed at 20 °C for 30 min, 65 °C for 30 min and 4 °C. Then, 30 µl NEBNext Ultra II Ligation Master Mix, 1 µl NEBNext Next Ligation Enhancer and 2.5 µl NEBNext Adaptor (diluted according to the initial amount of nucleic acid) were added to the product and incubated at 20 °C for 15 min. Then, 3 µl USER Enzyme was added and incubated at 37 °C for 15 min. After double screening with 0.9 × magnetic beads (according to the initial amount), the beads were washed twice with 80% ethanol and suspended in 20 µl buffer EB (Qiagen). Next, 15 µl of product was mixed with 5 µl index i7/i5 and 25 µl NEBNext Ultra II Q5 Master Mix, and run according to the following PCR program: 98 °C for 30 s; 98 °C for 10 s; 65 °C for 75 s (the number of cycles is set according to the initial amount of nucleic acid); 65 °C for 5 min; and 4 °C hold. The library was recovered with the same amount of magnetic beads and washed twice with 80% ethanol. Thirty-three microliters of EB buffer (Qiagen) was used to suspend the magnetic beads. Qubit, agarose gel electrophoresis and qPCR were used for quality control.

### mNGS

CSF DNA and RNA were used to construct the sequencing library, which was sequenced using Illumina Nova-seq (Illumina Inc., United States) by Novogene Technology Co., Ltd. (China). Approximately 700 GB of data was generated.

### Bioinformatics

The low-quality bases (quality value ≤ 30) and adapter sequences were trimmed using Trimmomatic with default parameters. The reads with fewer than 36 (samples with bacteria) or 140 (samples with viruses) were filtered out. Accordingly, clean data were obtained.

Kraken2 was used to classify sequencing data. Kraken2 exhibited similar, and often superior, per-sequence accuracy to the other nucleotide classifiers with high processing speed and fewer memory requirements [26]. Kraken2 classifies overlapping 31-kmer bp sequences by mapping them to the most recent common ancestor to provide the most accurate taxonomic classification, such as species and genus. The reference databases for Kraken2 were built from RefSeq bacteria, archaea, and viral libraries and the GRCh38 human genome (ftp://ftp.ccb.jhu.edu/pub/data/kraken2_dbs/). Kraken2 was used with default parameters. The results of Kraken2 were visualized and organized by Pavian v1.0 (The Center for Computational Biology at Johns Hopkins University). The details of the bioinformatics are shown in Fig. 1B.

### Analysis of results

The positive cutoff of nonviral pathogens was set as previously reported. If the reads per million (RPM) of a certain microorganism in CSF samples was greater than or equal to 10 times the RPM in the NEC negative control RPM ratio, the microorganism was identified as positive [11, 22]. If microorganisms were not detected in the negative cerebrospinal fluid, the RPM in NEC was set to 1, and then the RPM ratio was calculated. The positive viral cutoff was three noncontiguous or nonoverlapping fragments of more than 140 bp on the genome covered, and the viral species did not exist in the NEC [10]. The coverage was displayed by Integrative Genomics Viewer (IGV_2.8.10., The IGV Team is based at UC San Diego and the Broad Institute of MIT and Harvard).

### Definitions and calculation formula

RR: Raw reads, refers to the number of reads obtained after Kraken2 classification to a specific taxon.

GS: Genome size, refers to the size of the microorganism’s genome (Mb).

NR: Normalized reads, refers to standardized read numbers obtained by normalizing the raw reads (RR) in each sample according to the size of the microorganism's genome. $\mathrm{NR}=\frac{RR}{\mathrm{GS}\times {10}^{3}}$

TR: Total reads, refers to the total reads of a sample.

RPM_sample_: The RPM of a certain microorganism in cerebrospinal fluid samples. ${RPM}_{sample}=\frac{{{10}^{6}\times RR}_{sample }}{{TR}_{sample}}$

RPM_NEC_: The RPM of a certain microorganism in NEC. ${RPM}_{NEC}=\frac{{{10}^{6}\times RR}_{NEC }}{{TR}_{NEC}}$

RPM_ratio_: The ratio of RPM _sample_ to RPM _NEC_. If RR_NEC_ ≠ 0, ${RPM}_{ratio}=\frac{{RPM}_{sample }}{{RPM}_{NEC}}$.

If RR_NEC_ = 0, RPM_NEC_ = 1 and ${RPM}_{ratio}=\frac{{RPM}_{sample }}{1}$

Coverage percent: The ratio of base sites covered by more than ten valid reads to genomic length. The number of base sites covered by more than ten valid reads is supposed to be N. $\mathrm{Coverage percent}=\frac{N}{\mathrm{GS}\times {10}^{6}}$

### Statistical analysis

Multiple comparison corrections for P values were performed by p.adjust command in R using 'fdr' method.

### Clinical CSF collection

In the case of clinical deemed necessary, inpatients were subjected to lumbar puncture to obtain CSF. After completing the tests required by the clinician, the remaining CSF were collected. The remaining CSF after laboratory testing was collected. This procedure was performed in accordance with the Declaration of Helsinki with regard to ethical principles for research involving using human specimens and approved by the Ethics Committee of Shenzhen Children’s Hospital (2017027). CSF samples were stored at −80 °C.

## Results

### NEB produced more high-quality reads than Illumina

**Fig. 2:**
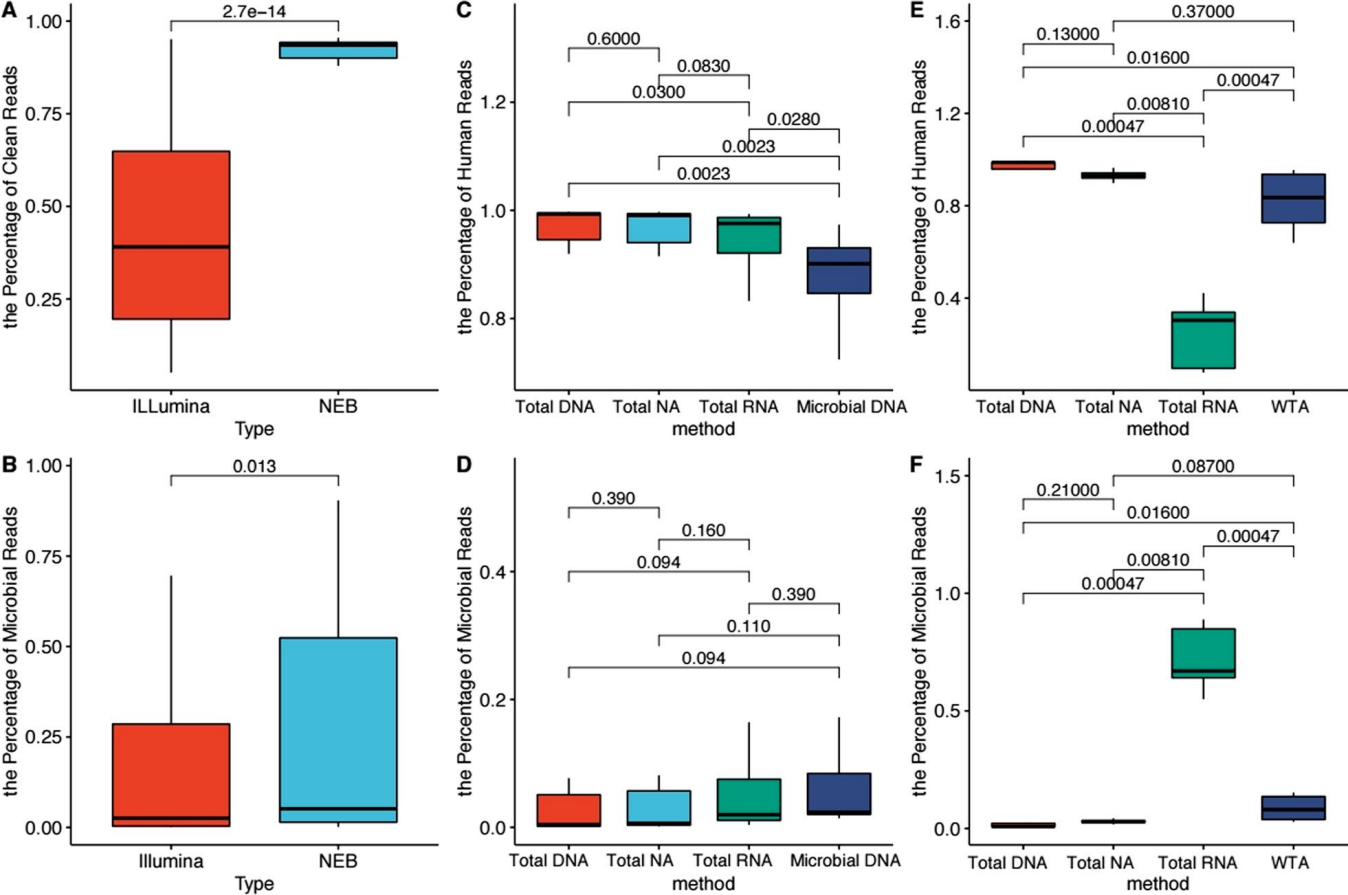
Comparison of clean reads, human reads and microbial reads. **A** Proportion of clean reads to raw reads by Illumina or NEB. **B** Proportion of microbial reads to raw reads by Illumina or NEB. **C** Human reads in sample 1–9 of Microbial DNA, Total DNA, Total NA and Total RNA. **D** Microbial reads in sample 1–9 of Microbial DNA, Total DNA, Total NA and Total RNA. **E** Human reads in sample 10–13 of Total DNA, Total NA, Total RNA and WTA. **F** Microbial reads in sample 10–13 of Total DNA, Total NA, Total RNA and WTA

Sequencing information of each sample is shown in Additional file 2: Table S2. After raw reads were trimmed, the clean reads were further classified into four compositions by Kraken2: human, non-virus, virus and unclassified reads. Illumina and NEB are commonly used kits. We compared the clean read percentage (clean reads/total raw reads) (Fig. 2A) and microbial read percentage (microbial reads/total raw reads) (Fig. 2B) after library generation. We found that the NEB method obtained more high-quality reads and microbial reads than Illumina (adjusted P = 2.7e−14 and adjusted P = 0.013).

### The proportion of pathogens is increased by microbial DNA and total RNA methods

More than 90% of the metagenomic reads of human samples belong to the human genome. Reducing host DNA contamination can enrich the proportion of pathogens. For samples with exogenous addition of SP or *E. coli*, we compared the ability of microbial DNA, total DNA, total NA and total RNA to enrich the proportion of microbial reads (Fig. 2C and D). As seen in Fig. 2C, Microbial DNA extraction significantly reduced the percentage of human read population in comparison with total DNA (adjusted P = 0.0023), total NA (adjusted P = 0.0023) and total RNA (adjusted P = 0.028).

For samples with exogenous addition of EBV or EV71, we compared the ability of total NA, total DNA, total RNA and WTA to enrich the proportion of microbial reads (Fig. 2E and F). Total RNA extraction led to a significantly lower proportion of human reads compared with total NA (adjusted P = 0.00810), WTA (adjusted P = 0.00047) and total DNA (adjusted P = 0.00047) (Fig. 2E) and a significantly higher proportion of microbial reads compared with total NA (adjusted P = 0.00810), WTA (adjusted P = 0.00047) and total DNA (adjusted P = 0.00047) (Fig. 2F). We also analyze EBV and EV71 separately (Additional file 4: Fig. S1). For EBV, Total RNA extraction led to a higher proportion of microbial reads compared with total NA, WTA and total DNA with the adjusted-P value were 0.057. For EV71,Total RNA extraction led to a significantly higher proportion of microbial reads compared with total NA and WTA (adjusted P = 0.029). WTA produced more microbial reads than total NA (adjusted P = 0.029).

Based on the above results, both microbial DNA and total RNA were effective in reducing human DNA contamination. DNaseI is able to eliminate human genomic DNA.

### Total NA and total RNA can detect all target pathogen types

**Fig. 3:**
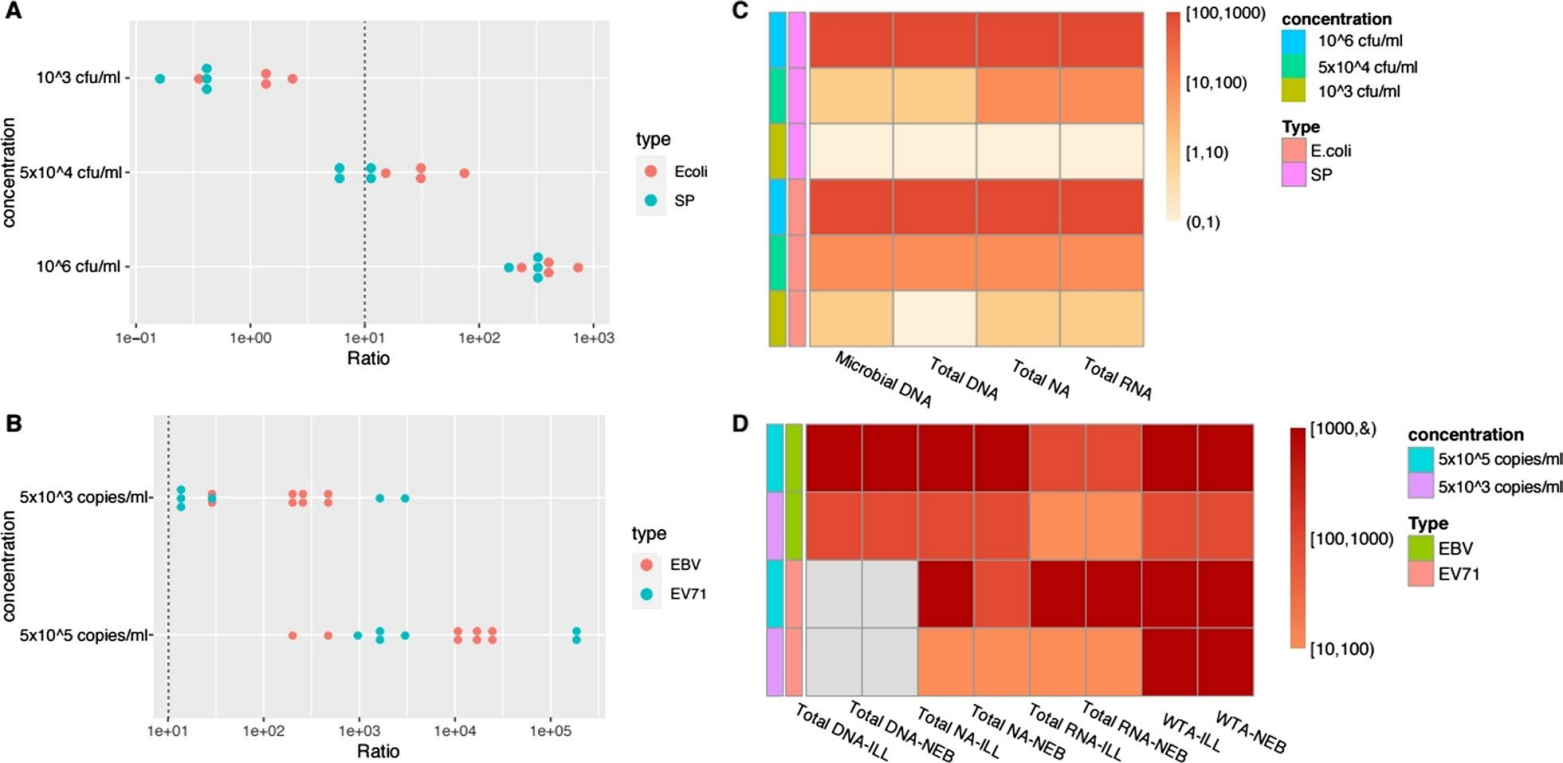
RPM_ratio_ of target pathogen in each sample. **A** RPM_ratio_ of SPN and E.coli. There were three concentration gradients in y-axis: 10^3^ CFU / ml, 5 × 10^4^ CFU / ml and 10^5^ CFU / ml. The red solid dots represent RPM_ratio_ of E.coli by microbial DNA, total DNA, total Na and total RNA. The blue solid dots represent RPM_ratio_ of SPN by microbial DNA, total DNA, total Na and total RNA. The dashed line highlights the threshold of ratio = 10. **B** RPM_ratio_ of EBV and EV71. There were two concentration gradients in y-axis: 5 × 10^3^ copies/ml, 5 × 10^5^ copies/ ml. The red solid dots represent RPM_ratio_ of EBV by Total DNA, Total NA, Total RNA and WTA. The blue solid dots represent RPMratio of EV71 by Total DNA, Total NA, Total RNA and WTA. **C** Heat map summarizing RPM_ratio_ of E. coli and SPN in sample 1–6. **D** Heat map summarizing RPM_ratio_ of EBV and EV71 in sample 10–13. The position of missing samples is filled with gray

For exogenous addition, we selected four representative encephalitis pathogens: gram-positive bacteria, SP; gram-negative bacteria, *E. coli*; enveloped DNA virus, EBV and nonenveloped RNA virus, EV71. According to the Kraken results and positive cutoff, all the microorganisms found in each sample are listed in Additional file 3: Table S3. The heat maps and scatter maps are based on the four added pathogens. As seen in Fig. 3A, E. *coli* was more abundant than SP, which may be due to the higher nucleic acid extraction efficiency of gram-negative bacteria. However, at a low concentration (10^3^ CFU/ml), the RPM_ratio_ of the two bacteria did not reach the positive threshold (10). Figure 3B shows that the RPM_ratio_ of EBV and EV71 under different processes are relatively discrete, indicating that pretreatment/nucleic acid extraction methods have an important influence on the detection of EBV and EV71. The RPM_ratio_ of EBV and EV71 exceeded 10 at 10^3^ copies/ml and 10^5^ copies/ml. Both total NA and total RNA detected all the added pathogens: SPN, *E. coli*, EBV and EV71 (Fig. 3C and D).

### Different types of microorganisms respond better to specific pretreatment/nucleic acid extraction methods

**Fig. 4:**
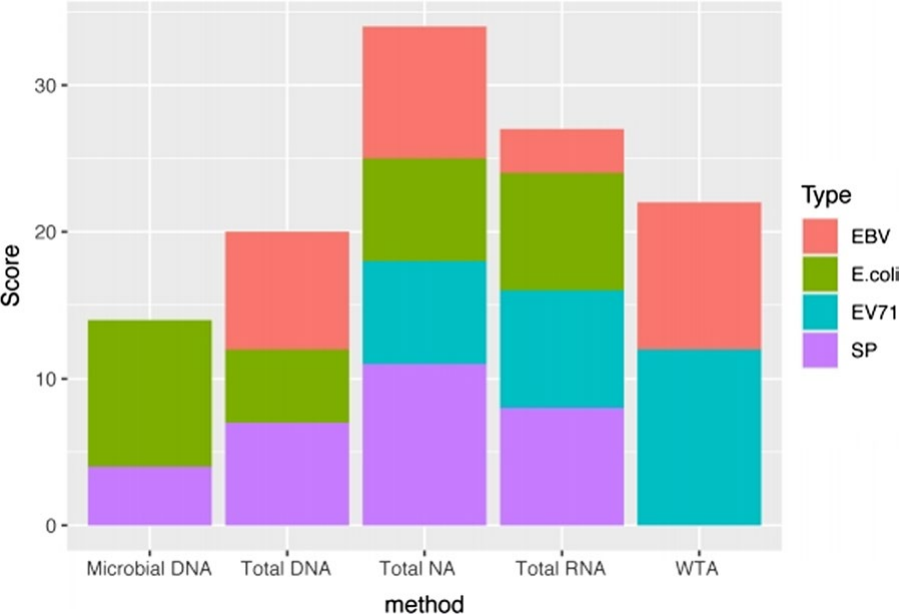
Grading of five pretreatment / nucleic acid extraction methods. The total scores of microbial DNA, total DNA, total NA, total RNA and WTA were compared. The respective scores corresponding to four target pathogens were represented by respective colored columns

To be objective and comprehensive, we set a scoring system to estimate the five pretreatment/nucleic acid extraction methods of total DNA, total NA, total RNA, microbial DNA and WTA. The NR, RPM_ratio_ and coverage percentage of target pathogens were taken as scoring indexes (Additional file 4: Fig. S2). NR refers to normalized reads. RPM_ratio_ represents the specific abundance of pathogens excluding background noise. The higher coverage percentage reflects that the number of specific reads is large and genomic coverage is relatively uniform. We ranked each index from highest value to lowest value and gave 4, 3, 2, 1, and 0 points, respectively. The indexes were separately scored and then summed as the final score of a method. We calculated the scores of five sample pretreatment/nucleic acid extraction methods and illustrated them in Fig. 4. Microbial DNA extraction has the strongest detection ability for *E. coli*, while total NA exhibits the best SP detection ability. Viruses are more easily detected using the WTA method. For all four pathogens, the total NA method ranked the highest, followed by total RNA. Total NA and total RNA methods can detect representative pathogens of encephalitis and benefit the diagnosis of infectious encephalitis with unknown etiology.

### Pathogens of encephalitis can be detected by total NA

Five inpatients with infectious encephalitis were enrolled. They were clinically diagnosed with *Streptococcus agalactiae* encephalitis (1), *Acinetobacter baumannii* encephalitis (1), HSV-1 encephalitis (1) and enterovirus encephalitis (2). Cerebrospinal fluid samples were obtained from a biobank. Total nucleic acids were extracted by the total NA method, and libraries were generated using an NEB kit. The clinical etiology and mNGS results are listed in Table 1. The results showed that the total NA method could detect gram-positive bacteria, i.e., *Streptococcus agalactiae*, gram-negative bacteria, i.e., *Acinetobacter baumannii*, DNA virus HSV-1, RNA virus echovirus 30 and RNA virus coxsackievirus B5 in cerebrospinal fluid. The genomic coverage maps of echovirus 30 and coxsackievirus B5 in samples 0006 and 0010 are shown in Additional file 4: Fig. S3A and B, respectively.

**Table 1:** Infections diagnosed by Metagenomic NGs with “Total NA extraction”

Sample ID	mNGs result with total NA	Clinical etiology	
		Result	Method
0001	Herpes simplex virus type 1	Herpes simplex virus type 1	Fluorescent quantitative PCR of CSF
0004	Acinetobacter baumannii (0.68)* Acinetobacter pittii (0.29) Acinetobacter calcoaceticus(0.18)	Acinetobacter baumannii	Bacterial culture of CSF
0006	Echovirus 30	Enterovirus	Fluorescent quantitative PCR of CSF
0008	Streptococcus agalactiae	Streptococcus agalactiae	Bacterial culture of CSF
0010	Coxsackievirus B5	Enterovirus	Fluorescent quantitative PCR of CSF

^*^The corresponding genomic coverage of pathogen species is shown in brackets

## Discussion

Next-generation sequencing and metagenomics help to diagnose acute infectious encephalitis with unknown etiology. The promoted technology and lowered cost benefits clinical application. In order to detect DNA and RNA pathogens in cerebrospinal fluid, the existing strategy is to divide a sample into two parts, one extract DNA for DNA library and the other extract RNA for RNA library [20]. In our work, we established a mNGS protocol with a single extraction step (total NA) followed by a single library generation (NEB), which saves on time and costs. Our mNGS process is capable of detecting all pathogens in CSF, including DNA and RNA microorganisms.

We found that the effectiveness of sample pretreatment and nucleic acid extraction determined the sensitivity of mNGS and varied by specimen or pathogen. First, sample pretreatment affected the nucleic acid yield. Bead beating or lysozyme was used in sample pretreatment to destroy cell walls. Bead beating mixes glass beads with the sample and vibrates at high speed. Lysozyme, also known as muramidase or N-acetylmuramide glycanhydrolase, can decompose mucopolysaccharides and dissolve the bacterial cell wall [27]. The RPM_ratio_ of *SP* or *E.coli* was higher after bead beating than after lysosome treatment, indicating that bead beating has more force to destroy bacterial cell wall of gram-positive bacteria. Second, nucleic acid extraction is crucial in virus detection. EV71 can be detected by the total NA and total RNA methods. The RPM_ratio_ of EV71 by the total RNA method was higher than that by the total NA method. This may be because DNaseI degraded the host genome, thus increasing the proportion of EV71 nucleic acids. EBV can be detected by the total NA and total RNA methods. The RPM_ratio_ of EBV detected by the total RNA method was approximately one tenth of that detected by the total NA method. These results suggested that both viral particles and free mRNA were contained in the supernatant from EBV-producing cells. DNaseI digested the EBV genome via the RNA method and decreased the detected nucleic acids.

There is no standard process of metagenomics library generation. The Illumina Nestera XT Library Prep kit and NEBNext Ultra TM II DNA Library Prep Kit are widely used commercial kits. In our work, we found that the quality of reads produced by NEB was better than that produced by Illumina. The amount of nucleic acid in CSF was far less than the 1 ng required for the Illumina kit. The Illumina transposon enzyme may cut nucleic acids into smaller fragments, leading to lower-quality reads. Ultrasonic interruption was performed using an NEB kit. The fragments were purified by magnetic beads; thus, the produced reads were of higher quality.

In a study in 2019, Miller et al. evaluated the accuracy of mNGS diagnosis of 95 cases of encephalitis by drawing ROC curves of different RPM_ratio_ [20]. The previous result showed that detective accuracy was maximized when the RPM_ratio_ was 10. The positive cutoff of RPM_ratio_ for bacteria, fungi or parasites in CSF was 10. We continued to use the positive cutoff in our work. More than one microorganism met the positive cutoff in some samples. However, as CSF is a normally sterile site, microorganism coinfections are relatively causative for cases of meningitis or encephalitis. Therefore, we supplemented coverage percent to exclude falsely positive results. The coverage percentage was defined as the ratio of base sites covered by more than ten valid reads to genomic length. The most dominant bacteria in one sample must have the highest coverage percentage. For example, the coverage percentages of *Streptococcus pneumoniae*, *Streptococcus thermophilus* and *Mogibacterium diversum* were 0.9, 0.018 and 0.054, respectively, in sample Microbial DNA-ILL-1. According to the coverage map, the reads of *Streptococcus pneumoniae* were evenly distributed in the genome, while reads of *Streptococcus thermophilus* or *Mogibacterium diversum* were distributed in the genome with bias (Additional file 4: Fig. S4); thus, neither ST nor MD were truly positive pathogens. The coverage percentage helped to identify and eliminate the classification errors caused by homologous sequences among species. We also found that the coverage percentage of truly positive pathogens varied in distinguished samples or concentrations (Additional file 3: Table S3). We suggested that coverage percentage can only be used for comparison between different pathogens in the same sample but not for comparison between different samples of the same pathogen.

For the examination of viruses, we followed the positive criteria previously reported [10]: there were three discontinuous or nonoverlapping fragments with lengths greater than 140 bp in the genome. In our work, we found some nontarget pathogens that also met the positive cutoff, such as *Mycoplasma hyopneumoniae*, *Torque teno virus* and phages. *Mycoplasma hyopneumoniae* normally colonizes the respiratory tract of pigs and cannot cause human diseases [28]; Bacteriophages can only invade bacteria [29]; and *Torque teno virus* belongs to the normal flora of humans [30]. The pathogenic characteristics indicated that they were all false positives. We should combine the positive cutoff and pathogenicity of the microorganism to make a diagnosis.

In our work, we used the standard Kraken2 database to classify the sequencing reads and distinguished most of the target pathogens. However, the detection process of enteroviruses is special: Sample 12 and sample 13 contained the EV71 BrCr strain of enteroviruses. After the first Kraken classification, no reads mapped to the reference genome of EV71 in any subsample of 12 or 13. There were 10^2^–10^5^ reads mapped to the reference genome named human enterovirus (taxid = 1193974). However, the mapped reads were distributed overlappingly in the 200–700 bp region of the genomic 5' end, which did not meet the positive criteria of the viruses. Therefore, we added a reference genome of EV71 (taxid = 69153) to the database and conducted the second classification. EV71 was then successfully detected in all subsamples. We explained that the reason for missed EV71 by mNGS was that the reference genome of EV71 was absent. We checked that there were only 13 reference genome sequences of enterovirus strains in the Kraken2 standard database, and some epidemic enteroviruses, such as EV71, CA16 and echovirus, were excluded. Thus, we supplemented all the enterovirus genomic sequences published in NCBI. Echovirus 30 and coxsackievirus B5 were detected in clinical sample 0006 and clinical sample 0010 by blasting to the new database. The metagenomic database determined the sensitivity and specificity of pathogen detection. Common pathogenic microorganisms, clinically rare pathogens and newly emerging pathogens should be included to improve the sensitivity of mNGS, while nonpathogenic microorganisms such as bacteriophages and environmental microorganisms should be excluded to eliminate false positives and enhance specificity.

We suggest that the total NA method combined with the NEB library generation kit is the optimal procedure for cerebrospinal fluid pathogen detection. We also suggest setting up the appropriate negative controls, positive controls and internal quality controls of the metagenomic experiments. Additionally, we have launched a clinical research project to evaluate the sensitivity, specificity and accuracy of our in-house mNGS diagnosis.

## Conclusions

We established a mNGS protocol with a single extraction step (total NA) followed by a single library generation (NEB), which is effective in detecting both DNA and RNA pathogens in CSF. The protocol was applied to diagnose encephalitis in children and the results were consistent with etiological methods. The advantages of our protocol are as below: Firstly, the strategy covering DNA and RNA pathogens is helpful to diagnose infection without any hypothesis. Secondly, the protocol saves time and reduces costs of wet experiment and sequencing. Clinicians and patients prefer efficient and low-cost diagnostic methods. The last point is that we set up strict positive cutoffs to remove false positives. For example, both RPM_ratio_ and coverage percentage are used to determine positive bacteria. However, the imperfect metagenomic database leads to missed or wrong results of positive pathogens. A specific genome database of pathogens causing human diseases will make up for this shortcoming.

## Supplementary Information

**Additional file 1: Table S1.** Sample ID and Pathogens contained.

**Additional file 2: Table S2.** Sequencing information of samples with different library preparation methods and pathogens.

**Additional file 3: Table S3.** Metagenomic sequencing results of each sample.

**Additional file 4: Fig. S1.** The microbial reads percentage of EBV samples and EV71 samples. A. The microbial reads percentage of EBV samples in methods Total DNA , Total NA ,Total RNA and WTA. B. The microbial reads percentage of EV71 samples in methods Total NA ,Total RNA and WTA. **Fig. S2.** NR, RPM_ratio_ and Coverage percent of target pathogens. Subfigure A/B/C shows NR/ RPM_ratio_/coverage percentage of E. coli respectively comparing four sample pretreatment/nuclear acid extraction methods including Microbial DNA, Total DNA, Total NA and Total RNA. The red dots represent concentration of 10^6^cfu/ml and the blue dots represent concentration of 5x10^4^ cfu/ml. Subfigure D/E/F shows NR/ RPM_ratio_/coverage percentage of SP comparing four sample pretreatment/nuclear acid extraction methods respectively. The red dots represent concentration of 10^6^cfu/ml and the blue dots represent concentration of 5x10^4^ cfu/ml. Subfigure G/H/I shows NR/RPM_ratio_/coverage percentage of EBV respectively comparing four sample pretreatment/nuclear acid extraction methods including Total DNA, Total NA, Total RNA and WTA. The red dots represent concentration of 5x10^5^ copies/ml and the blue dots represent concentration of 5x10^3^ copies/ml. Subfigure J/K/L shows NR/RPM_ratio_/coverage percentage of EV71 respectively comparing four sample pretreatment/nuclear acid extraction methods respectively. The red dots represent concentration of 5x10^5^ copies/ml and the blue dots represent concentration of 5x10^3^ copies/ml. **Fig. S3.** Genomic coverage of pathogens in CSF from encephalitis patients. A. Genomic coverage of Echovirus 30 in clinical CSF 0006. B. Genomic coverage of coxsackievirus B5 in clinical CSF 0010. **Fig. S4.** Genomic coverage of *Streptococcus pneumoniae*, *Streptococcus thermophilus* and* Mogibacterium diversum* in sample Microbial DNA-ILL -1. A. Genomic coverage of *Streptococcus pneumoniae*. B. Genomic coverage of *Streptococcus thermophilus*. C. Genomic coverage of *Mogibacterium diversum*.

## Data Availability

All the sequencing data generated in this study was deposited in SRA with accession number PRJNA752873.

## Abbreviations

mNGs: Metagenomic next-generation sequencing
CSF: Cerebrospinal fluid
SP: *Streptococcus pneumoniae*
E. coli: *Escherichia coli*
EBV: Epstein Barr virus
EV71: Enterovirus 71
NEC: Negative extraction control
