# Supplementary material for: Comparison and development of a metagenomic next-generation sequencing protocol for combined detection of DNA and RNA pathogens in cerebrospinal fluid
Source: BMC Infect Dis. 2022 Apr 1;22:326. doi: 10.1186/s12879-022-07272-y (PMC8976360; doi:10.1186/s12879-022-07272-y)
Supplement: Supplementary file 1 — Additional file 1: Table S1. Sample ID and Pathogens contained. [file 12879_2022_7272_MOESM1_ESM.docx]

**Supplemental Table 1 . Sample ID and Pathogens contained**

| No. of sample | Pathogens  contained | Titer  （cfu/ml or copies/ml） | No. of sub-samples | | | | | | | | | |
| --- | --- | --- | --- | --- | --- | --- | --- | --- | --- | --- | --- | --- |
|  |  |  | **Microbial DNA-ILL** | **Microbial DNA-NEB** | **Total DNA-ILL** | **Total DNA-NEB** | **Total NA-ILL** | **Total**  **NA-NEB** | **Total**  **RNA-ILL** | **Total**  **RNA-NEB** | **WTA-ILL** | **WTA-NEB** |
| 1 | Spn | 10^6^ | **Microbial DNA-ILL** -1 | **Microbial DNA-NEB** -1 | **Total DNA-ILL** -1 | **Total DNA-NEB** -1 | **Total NA-ILL** -1 | **Total**  **NA-NEB** -1 | **Total**  **RNA-ILL** -1 | **Total**  **RNA-NEB** -1 | — | — |
| 2 |  | 5 X 10^4^ | **Microbial DNA-ILL** -2 | **Microbial DNA-NEB** -2 | **Total DNA-ILL** -2 | **Total DNA-NEB** 2 | **Total NA-ILL** -2 | **Total**  **NA-NEB** -2 | **Total**  **RNA-ILL** -2 | **Total**  **RNA-NEB** 2 | — | — |
| 3 |  | 10^3^ | **Microbial DNA-ILL** -3 | **Microbial DNA-NEB** -3 | **Total DNA-ILL** -3 | **Total DNA-NEB** -3 | **Total NA-ILL** -3 | **Total**  **NA-NEB** -3 | **Total**  **RNA-ILL** -3 | **Total**  **RNA-NEB** -3 | — | — |
| 4 | E. coli | 10^6^ | — | **Microbial DNA-NEB** -4 | **Total DNA-ILL** -4 | **Total DNA-NEB** -4 | **Total NA-ILL** -4 | **Total**  **NA-NEB** -4 | **Total**  **RNA-ILL** -4 | **Total**  **RNA-NEB** -4 | — | — |
| 5 |  | 5 X 10^4^ | — | **Microbial DNA-NEB** -5 | **Total DNA-ILL** -5 | **Total DNA-NEB** -5 | **Total NA-ILL** -5 | **Total**  **NA-NEB** -5 | **Total**  **RNA-ILL** -5 | **Total**  **RNA-NEB** -5 | — | — |
| 6 |  | 10^3^ | — | **Microbial DNA-NEB** -6 | **Total DNA-ILL** -6 | **Total DNA-NEB** -6 | **Total NA-ILL** -6 | **Total**  **NA-NEB** -6 | **Total**  **RNA-ILL** -6 | **Total**  **RNA-NEB** -6 | — | — |
| 7* | Spn + E. coli | 10^6^ | — | **Microbial DNA-NEB** -7 | **Total DNA-ILL** -7 | **Total DNA-NEB** -7 | **Total NA-ILL** -7 | **Total**  **NA-NEB** -7 | **Total**  **RNA-ILL** -7 | **Total**  **RNA-NEB** -7 | — | — |
| 8* |  | 5 X 10^4^ | — | **Microbial DNA-NEB** -8 | **Total DNA-ILL** --8 | **Total DNA-NEB** -8 | **Total NA-ILL-** 8 | **Total**  **NA-NEB** -8 | **Total**  **RNA-ILL** -8 | **Total**  **RNA-NEB** -8 | — | — |
| 9* |  | 10^3^ | — | **Microbial DNA-NEB** -9 | **Total DNA-ILL** 9 | **Total DNA-NEB** -9 | **Total NA-ILL-** 9 | **Total**  **NA-NEB** -9 | **Total**  **RNA-ILL** -9 | **Total**  **RNA-NEB** -9 | — | — |
| 10 | EBV | 5 X 10^5^ | — | — | **Total DNA-ILL** -10 | **Total DNA-NEB** -10 | **Total NA-ILL** -10 | **Total**  **NA-NEB** -10 | **Total**  **RNA-ILL** -10 | **Total**  **RNA-NEB** -10 | **WTA-ILL** -10 | **WTA-NEB -**10 |
| 11 |  | 5 X 10^3^ | — | — | **Total DNA-ILL** -11 | **Total DNA-NEB** -11 | **Total NA-ILL** -11 | **Total**  **NA-NEB** -11 | **Total**  **RNA-ILL** -11 | **Total**  **RNA-NEB** -11 | **WTA-ILL** -11 | **WTA-NEB** -11 |
| 12 | EV71 | 5 X 10^5^ | — | — | — | — | **Total NA-ILL** -12 | **Total**  **NA-NEB** -12 | **Total**  **RNA-ILL** -12 | **Total**  **RNA-NEB** -12 | **WTA-ILL** -12 | **WTA-NEB** -12 |
| 13 |  | 5 X 10^3^ | — | — | — | — | **Total NA-ILL** -13 | **Total**  **NA-NEB** -13 | **Total**  **RNA-ILL** -13 | **Total**  **RNA-NEB** -13 | **WTA-ILL** -13 | **WTA-NEB** -13 |
| Negative control | | | **Microbial DNA-ILL** -N | **Microbial DNA-NEB** -N | **Total DNA-ILL** -N | **Total DNA-NEB** -N | **Total NA-ILL** -N | **Total**  **NA-NEB** -N | **Total**  **RNA-ILL** -N | **Total**  **RNA-NEB** -N | **WTA-ILL** -N | **WTA-NEB** -N |

* The content ratio of Streptococcus pneumoniae to Escherichia coli in these samples is 3: 1.

— not done
