# Supplementary material for: Comparison and development of a metagenomic next-generation sequencing protocol for combined detection of DNA and RNA pathogens in cerebrospinal fluid
Source: BMC Infect Dis. 2022 Apr 1;22:326. doi: 10.1186/s12879-022-07272-y (PMC8976360; doi:10.1186/s12879-022-07272-y)
Supplement: Supplementary file 3 — Additional file 3: Table S3. Metagenomic sequencing results of each sample. [file 12879_2022_7272_MOESM3_ESM.docx]

**Supplementary Table 3. Metagenomic sequencing results of each sample**

| Non-viral  Pathogen | Methods  Sample | Microbial DNA-ILL | Microbial DNA-NEB | Total DNA-ILL | Total DNA-NEB | Total NA-ILL | Total NA-NEB | Total RNA-ILL | Total RNA-NEB |
| --- | --- | --- | --- | --- | --- | --- | --- | --- | --- |
|  | 1 | SPN(0.9)  MD(0.018)  ST(0.054) | SPN(0.94) | SPN(0.92) | SPN(0.94) | SPN(0.91) | SPN(0.94)  AB(0.049) | SPN(0.73) | SPN(0.92)  AB(0.17)  KP (0.098) |
|  | 2 | SPN(0.27) | _ | SPN(0.051) | _ | SPN(0.017) | SPN(0.33)  AB(0.048) | SPN(0.02) | SPN(0.26)  AB(0.17)  KP(0.06) |
|  | 3 | _ | _ | _ | _ | _ | AB(0.049) | _ | AB(0.24)  KP(0.12) |
|  | 4 |  | Ecoli(0.71) | Ecoli(0.72) | Ecoli(0.71) | Ecoli(0.69)  SE(0.069) | Ecoli(0.72)  BA(0.009)  AB(0.06)  SE(0.11)  KP(0.08) | Ecoli(0.54)  SE(0.052) | Ecoli(0.67)  AB(0.24)  KP(0.14)  SE(0.09)  BA(0.009)  CBC(0.01)  CHE(0.008) |
|  | 5 |  | Ecoli(0.68) | Ecoli（0.52） | Ecoli(0.67) | Ecoli (0.22) | Ecoli (0.59)  AB(0.16)  KP(0.08) | Ecoli(0.67) | Ecoli(0.3)  AB(0.06)  KP(0.044) |
|  | 6 |  | _ | _ | _ | _ | AB(0.032) | _ | AB(0.33)  KP(0.099) |
|  | 7 | SPN （0.79）  SA （0.90） | Ecoli(0.71)  SPN(0.92) | SPN(0.89)  Ecoli（0.69） | SPN (0.94)  Ecoli（0.71） | SPN(0.75)  Ecoli(0.12) | SPN(0.94)  Ecoli(0.71)  AB(0.018) | SPN(0.66)  Ecoli(0.21) | SPN(0.92)  Ecoli(0.65)  AB(0.19)  KP(0.099)  SE(0.08)  BC(0.04) |
|  | 8 | SPN（0.22）  SA（0.58）  SR（0.02） | _ | _ | _ | SPN(0.014) | AB(0.044) | _ | Ecoli(0.18)  AB(0.25)  KP(0.1) |
|  | 9 | _ | _ | _ | _ | _ | AB(0.092) | _ | AB(0.22)  KP(0.11) |

The table lists the positive results of nonviral samples 1-9. All microorganisms in each sample that meet the positive cutoff of nonviral pathogens are listed in the corresponding grid and followed by its coverage percent value.

- indicates the sample contains no positive microorganism

Gray filled grids are missing samples

The corresponding full name of the microbe abbreviation is as follows：SPN：Streptococcus pneumoniae Ecoli：Escherichia coli MD：Mogibacterium diversum ST：Streptococcus thermophilus SA：Staphylococcus aureus SR：Stenotrophomonas rhizophila SE：Salmonella enterica AB：Acinetobacter baumannii BA：Buchnera aphidicola KP：Klebsiella pneumoniae CBC：Candidatus Baumannia cicadellinicola CHE:Candidatus Hoaglandella endobia BC: Bacillus cereus

| Virus | Method  Sample | Total DNA-ILL | Total DNA-NEB | Total NA-ILL | Total NA-NEB | Total RNA-ILL | Total RNA-NEB | WTA-ILL | WTA-NEB |
| --- | --- | --- | --- | --- | --- | --- | --- | --- | --- |
|  | 10 | EBV | EBV | EBV | TTV15  EBV | EBV | EBV  Lactococcus virus Bibb29  Lactococcus phage phi7 | EBV | EBV  TTV  TTV-19 |
|  | 11 | EBV | EBV | EBV  TTV15 | EBV | EBV | EBV | EBV | EBV |
|  | 12 |  |  | EV71 | EV71  MH | EV71 | Enterobacteria_phage_cdtI  Enterobacteria_phage_YYZ-2008  EV71  MH  Salmonella_phage_RE_2010 | EV71 | EV71  MH |
|  | 13 |  |  | EV71  TTV15 | EV71  TTV15 | EV71 | Enterobacteria_phage_cdtI  Enterobacteria_phage_SfI  EV71  Ev-T1  Salmonella_phage_RE-2010 | EV71 | EV71  K13_Salmonella_virus_HK620 |

The table lists the positive results of viral samples 10-13. All microorganisms in each sample that meet the positive cutoff of viral pathogens are listed in the corresponding grid and followed by its coverage percent value.

- indicates the sample contains no positive microorganism

Gray filled grids are missing samples

The corresponding full name of the microbe abbreviation is as follows：EBV: Human gammaherpesvirus 4 TTV: Torque teno virus EV71: Human enterovirus 71 MH: Mycoplasma hyorhinis Ev-T1: Escherichia virus T1
