# Supplementary material for: Comparison and development of a metagenomic next-generation sequencing protocol for combined detection of DNA and RNA pathogens in cerebrospinal fluid
Source: BMC Infect Dis. 2022 Apr 1;22:326. doi: 10.1186/s12879-022-07272-y (PMC8976360; doi:10.1186/s12879-022-07272-y)
Supplement: Supplementary file 4 — Additional file 4: Fig. S1. The microbial reads percentage of EBV samples and EV71 samples. A. The microbial reads percentage of EBV samples in methods Total DNA , Total NA ,Total RNA and WTA. B. The microbial reads percentage of EV71 samples in methods Total NA ,Total RNA and WTA. Fig. S2. NR, RPMratio and Coverage percent of target pathogens. Subfigure A/B/C shows NR/ RPMratio/coverage percentage of E. coli respectively comparing four sample pretreatment/nuclear acid extraction methods including Microbial DNA, Total DNA, Total NA and Total RNA. The red dots represent concentration of 106cfu/ml and the blue dots represent concentration of 5x10^4^ cfu/ml. Subfigure D/E/F shows NR/ RPMratio/coverage percentage of SP comparing four sample pretreatment/nuclear acid extraction methods respectively. The red dots represent concentration of 106cfu/ml and the blue dots represent concentration of 5x10^4^ cfu/ml. Subfigure G/H/I shows NR/RPMratio/coverage percentage of EBV respectively comparing four sample pretreatment/nuclear acid extraction methods including Total DNA, Total NA, Total RNA and WTA. The red dots represent concentration of 5x10^5^ copies/ml and the blue dots represent concentration of 5x10^3^ copies/ml. Subfigure J/K/L shows NR/RPMratio/coverage percentage of EV71 respectively comparing four sample pretreatment/nuclear acid extraction methods respectively. The red dots represent concentration of 5x10^5^ copies/ml and the blue dots represent concentration of 5x10^3^ copies/ml. Fig. S3. Genomic coverage of pathogens in CSF from encephalitis patients. A. Genomic coverage of Echovirus 30 in clinical CSF 0006. B. Genomic coverage of coxsackievirus B5 in clinical CSF 0010. Fig. S4. Genomic coverage of Streptococcus pneumoniae, Streptococcus thermophilus and Mogibacterium diversum in sample Microbial DNA-ILL -1. A. Genomic coverage of Streptococcus pneumoniae. B. Genomic coverage of Streptococcus thermophilus. C. Genomic coverage of Mogibacterium diversum. [file 12879_2022_7272_MOESM4_ESM.docx]

**Supplemental Fig 1.The microbial reads percentage of EBV samples and EV71 samples.** A. The microbial reads percentage of EBV samples in methods Total DNA , Total NA ,Total RNA and WTA. B. The microbial reads percentage of EV71 samples in methods Total NA ,Total RNA and WTA.

**Supplemental Fig 2. NR, RPM_ratio_ and Coverage percent of target pathogens.** Subfigure A/B/C shows NR/ RPM_ratio_/coverage percentage of SPN respectively comparing four sample pretreatment/nuclear acid extraction methods including Microbial DNA, Total DNA, Total NA and Total RNA. The red dots represent concentration of 10^6^cfu/ml and the blue dots represent concentration of 5x10^4^ cfu/ml. Subfigure D/E/F shows NR/ RPM_ratio_/coverage percentage of E.coli comparing four sample pretreatment/nuclear acid extraction methods respectively. The red dots represent concentration of 10^6^cfu/ml and the blue dots represent concentration of 5x10^4^ cfu/ml. Subfigure G/H/I shows NR/RPM_ratio_/coverage percentage of EBV respectively comparing four sample pretreatment/nuclear acid extraction methods including Total DNA, Total NA, Total RNA and WTA. The red dots represent concentration of 5x10^5^ copies/ml and the blue dots represent concentration of 5x10^3^ copies/ml. Subfigure J/K/L shows NR/RPM_ratio_/coverage percentage of EV71 respectively comparing four sample pretreatment/nuclear acid extraction methods respectively. The red dots represent concentration of 5x10^5^ copies/ml and the blue dots represent concentration of 5x10^3^ copies/ml.


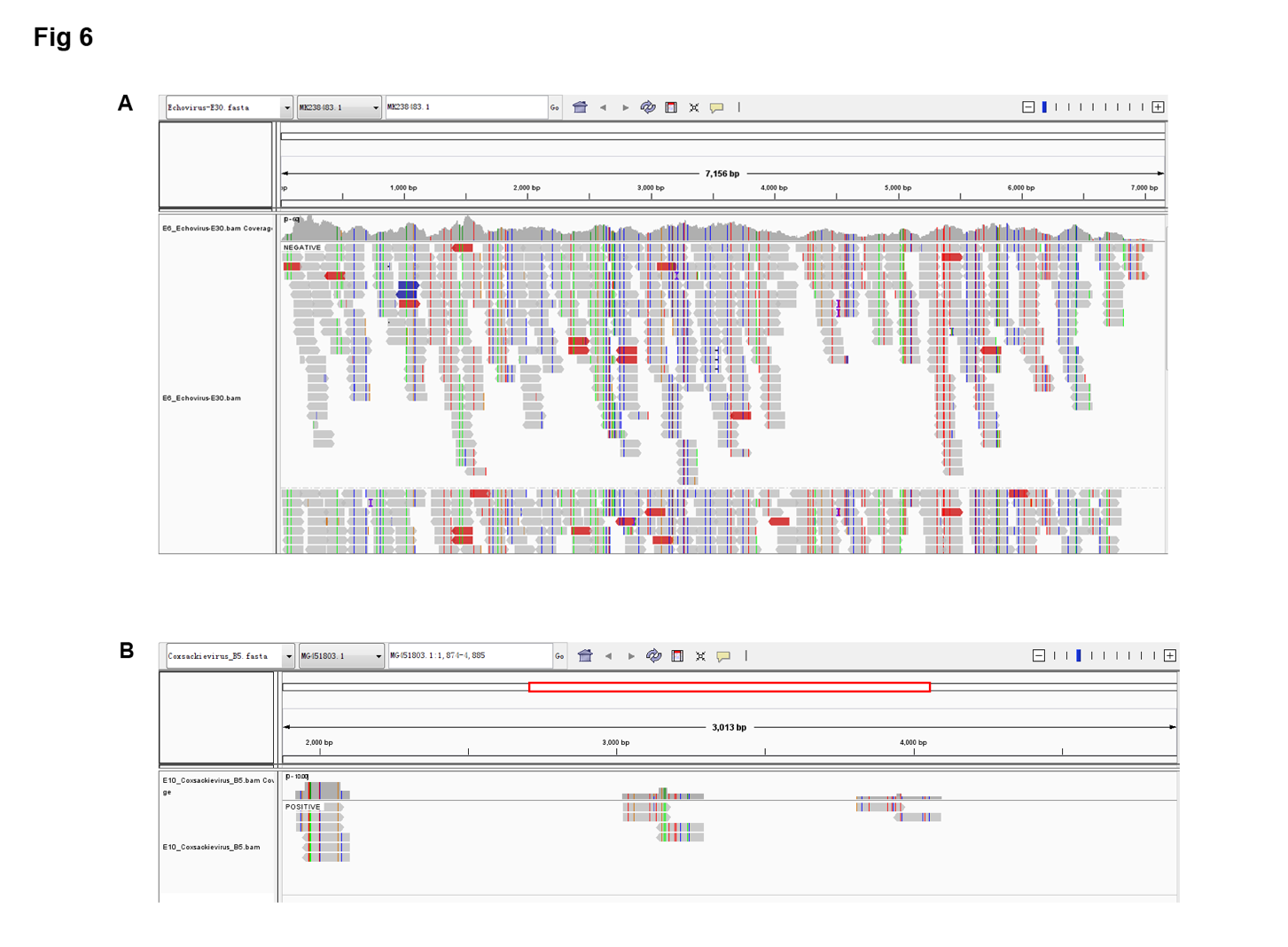


**Supplemental Fig 3. Genomic coverage of pathogens in CSF from encephalitis patients.** A. Genomic coverage of Echovirus 30 in clinical CSF 0006. B. Genomic coverage of coxsackievirus B5 in clinical CSF 0010.


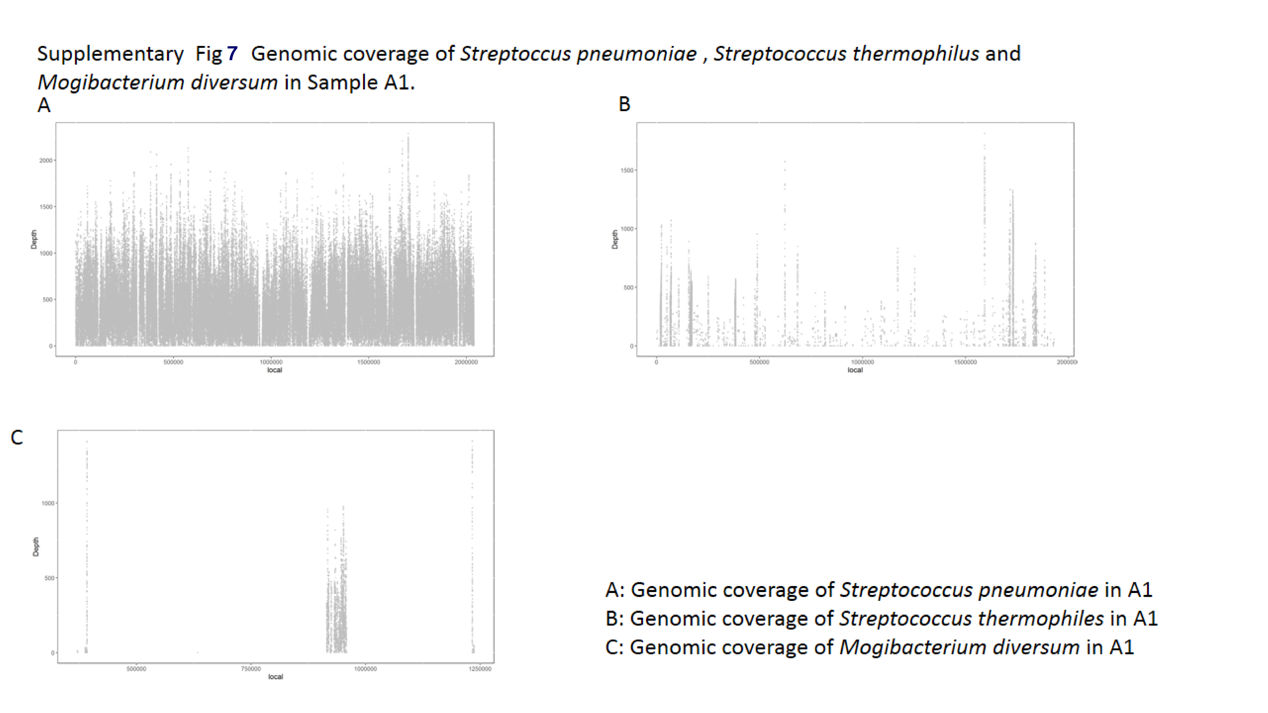


**Supplemental Fig 4. Genomic coverage of *Streptococcus pneumoniae*, *Streptococcus thermophilus* and *Mogibacterium diversum* in sample A1.** A. Genomic coverage of *Streptococcus pneumoniae* in sample A1. B. Genomic coverage of *Streptococcus thermophilus* in sample A1.C. Genomic coverage of *Mogibacterium diversum* in sample A1.
